# Scientific Challenges on Theory of Fat Burning by Exercise

**DOI:** 10.3389/fphys.2021.685166

**Published:** 2021-07-06

**Authors:** M. Brennan Harris, Chia-Hua Kuo

**Affiliations:** ^1^Department of Health Sciences, College of William and Mary, Williamsburg, VA, United States; ^2^Laboratory of Exercise Biochemistry, College of Kinesiology, University of Taipei, Taipei, Taiwan

**Keywords:** resistance training, fat loss, intensity, carbon and nitrogen redistribution theory, fatty acid oxidation, aerobic training, fat burner, obesity

## Abstract

Exercise training decreases abdominal fat in an intensity-dependent manner. The fat loss effect of exercise has been intuitively thought to result from increased fat burning during and after exercise, defined by conversion of fatty acid into carbon dioxide in consumption of oxygen. Nevertheless, increasing exercise intensity decreases oxidation of fatty acids derived from adipose tissue despite elevated lipolysis. The unchanged 24-h fatty acid oxidation during and after exercise does not provide support to the causality between fat burning and fat loss. In this review, alternative perspectives to explain the fat loss outcome are discussed. In brief, carbon and nitrogen redistribution to challenged tissues (muscle and lungs) for fuel replenishment and cell regeneration against abdominal adipose tissue seems to be the fundamental mechanism underlying the intensity-dependent fat loss effect of exercise. The magnitude of lipolysis (fatty acid release from adipocytes) and the amount of post-meal carbon and nitrogen returning to abdominal adipose tissue determines the final fat tissue mass. Therefore, meal arrangement at the time when muscle has the greatest reconstruction demand for carbon and nitrogen could decrease abdominal fat accumulation while increasing muscle mass and tissue repair.

## GRAPHICAL ABSTRACT

Exercise decreases abdominal fat mass, especially at high intensity. This outcome is not causally associated with fat burning, but better explained by carbon and nitrogen redistribution. Since abdominal fat tissue constantly releases fatty acids into circulation under post-absorptive condition with natural cell deaths, exercise diverts more post-meal carbon and nitrogen to muscle for energy repletion and cell regeneration after phagocytosis and stem cell homing. This in turn leads to concurrent fat mass loss and muscle mass gain. Respiratory ventilation during high-intensity aerobic exercise amplifies the competition for post-meal carbon and nitrogen against adipose tissues.

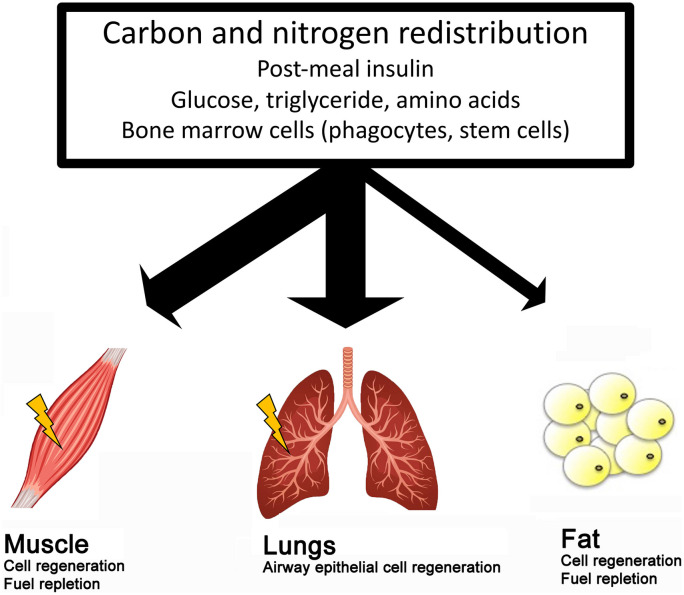

## The Scientific Challenges

Exercise training decreases abdominal fat, in which high-intensity exercise produces more prominent fat loss than low and moderate intensity exercise (Vissers et al., 2013; Viana et al., 2019). Fat burning is a classic theory to describe the abdominal fat-reducing outcome of exercise training. This theory is built on the intuition that exercise as an energy consuming behavior will increase fatty acid oxidation from abdominal fat stores compared with sedentary condition, and thus accounts for the fat loss outcomes of exercise training (Abbasi, 2019). Increased lipolysis with elevated circulating fatty acids together with increased oxygen consumption during exercise seems to favor this explanation (Romijn et al., 1993; Mora-Rodriguez and Coyle, 2000). However, the absolute energy contribution from plasma fatty acids (assuming all from adipose tissue) decreases as exercise intensity increases (from 25 to 85% VO_2max_) and is consistent with decreased tissue fatty acid uptake during exercise (Romijn et al., 1993). Furthermore, increased energy expenditure, especially during high intensity exercise, comes from fuel stored in skeletal muscle (mostly glycogen), not adipose tissue (fatty acids) (Romijn et al., 1993). Neither aerobic exercise nor resistance exercise increases 24-h fatty acid oxidation (Melanson et al., 2002).

A number of clinical studies divulges paradox between fat burning and fat loss outcome. A 15-weeks sprint training depending primarily on anaerobic metabolism effectively decreases abdominal fat, whereas moderate-intensity exercise training depending on aerobic metabolism with similar energy expenditure (60% VO_2max_ consuming ∼200 kcal, three times per week) failed to decrease body fat in young women (Trapp et al., 2008). Similarly, no fat loss effect was observed following 12-weeks of aerobic training at both low-intensity (40% VO_2max_) and moderate-intensity (70% VO_2max_) among obese men (∼350 kcal, three times per week) (Aggel-Leijssen et al., 2002). Therefore, an alternative theory to explain the fat loss outcome of exercise should be explored in order to provide robust scientific basis for designing effective fat loss training regimens.

Lipolysis appears to be more relevant with fat loss than fatty acid oxidation. Exercise increases plasma epinephrine levels at high intensities (Mora-Rodriguez and Coyle, 2000). Epinephrine stimulates lipolysis and inhibits the esterification of triglycerides via adrenergic receptors of adipocytes (Reilly et al., 2020), leading to release of free fatty acid from adipose tissue into circulation (Urhausen et al., 1994). Long-term adrenergic stimulation (i.e., clenbuterol and ractopamine) has been shown to decrease fat mass and increase muscle mass without changes in food intake and body temperature (Page et al., 2004). Abdominal adipocytes show much higher lipolytic response to epinephrine than gluteal adipocytes, which may partly explain the commonly observed abdominal fat loss response to high-intensity exercise training (Wahrenberg et al., 1989; Thompson et al., 2012).

The physiological significance of the enhanced release of fatty acids from lipolysis without the corresponding increase in fatty acid oxidation during and after exercise remains unclear. However, a proposed role of adipocyte-derived fatty acids in tissue repair has been recently described elsewhere (Shook et al., 2020). Fatty acids (e.g., eicosapentaenoate, linoleate, α-linolenate, γ-linolenate, and arachidonate) have been found to accelerate wound healing (Ruthig and Meckling-Gill, 1999). In addition, vascular structure formation can be enhanced by fatty acids, which is mediated by increasing reactive oxygen species and activating endothelial NOS synthase (Taha et al., 2020). Both findings implicate a possible role of elevated fatty acid concentrations in the repairing mechanism of exercise-induced tissue damage.

## Basic Assumption of Fat Burning Theory

The first basic assumption of fat burning theory is that fat cell death has no role in fat loss. However, this assumption is unlikely valid since fat cells are continuously dying and regenerating throughout our life. Approximately 8.4% of subcutaneous abdominal adipocytes are renewed annually with an average half-life of 8.3 years in human adults (Spalding et al., 2008). Abdominal fat mass is determined by the balance of fat cell death and regeneration of adipose tissue, which is influenced by exercise (Allerton et al., 2021). Acute adrenergic stimulation has been reported to induce fat cell death (Kim et al., 2010). The balance between fat cell death and regeneration is also strongly influenced by plasma insulin concentrations, which varies with exercise habit, meals, and sleeping fast. Lowering insulin for 2 weeks causes a massive fat loss >70%), associated with the death of adipocytes and endothelial cells in adipose tissues (Géloën et al., 1989). Lowering physical activity increases plasma insulin concentration and waist circumference without an observable change in body weight (Chen et al., 2006). In a contrast, high-intensity exercise lowers fasting and post-meal insulin levels while increasing the insulin sensitivity of exercised muscle (Ivy et al., 1999; Rice et al., 1999; Trapp et al., 2008), which partly explains the decreases in fat mass and increases in muscle mass among training individuals.

The second basic assumption of the fat burning theory is that muscle and fat cells are not interconvertible in a human body. However, we could not preclude the possibility that the fat mass loss concurrent with muscle mass gain after exercise training is associated with conversion between muscle and fat progenitor cells, derived from circulating bone marrow stem cells. Conversion from muscle satellite cells to an adipogenic lineage contributes the development of obesity and muscle mass loss in animals (Durschlag and Layman, 1983; Scarda et al., 2010). Glucose and reactive oxygen species (ROS) also stimulate the adipogenic conversion from muscle-derived stem cells (Aguiari et al., 2008). Both plasma glucose and ROS elevate with age and weight growth (Ho et al., 2019; Wang et al., 2019). However, exercise training lowers plasma glucose (Colberg et al., 2010) and ROS (Vinetti et al., 2015) in animals and humans. Circulating myokines released from contracting muscle also suppress adipogenesis and stimulate myogenesis (Barra et al., 2012; Ma et al., 2019). As a result, exercise appears to, at the very least, attenuate the conversion of muscle to fat and may instead activate the conversion of fat to muscle.

Further evidence of this mechanism comes from the wide array of exosomes (containing nucleic acids or peptide) released from exercising skeletal muscle implicating the crosstalk between muscle and fat tissues. Adipose tissues are a major source of circulating exosomes containing a variety of mediators, which may influence muscle development (Thomou et al., 2017; Ying et al., 2017). Some nucleic acid molecules encapsulated in the extracellular vesicles may play a role in the interconvertibility between fat and muscle progenitor cells. For example, muscle contraction induces acute increases of miR-21 into circulation (Xu et al., 2016). This molecule has been shown to inhibit proliferation of human adipose tissue-derived mesenchymal stem cells and high-fat diet-induced obesity (Kim et al., 2012). In addition, circulating miR-130 level has been found lowered in obese women and exercise stimulates release of miR-130 from skeletal muscle into circulation which inhibits adipogenesis (Lee et al., 2011).

The third assumption of the fat burning theory is that the increased carbon and nitrogen demands for airway epithelial cells regeneration in lungs does not contribute to fat loss during and after exercise. However, the possibility that the fat loss effect of high-intensity aerobic training due to competition for carbon and nitrogen between lungs and adipose tissues cannot be excluded (Leibacher and Henschler, 2016; Saat et al., 2016). The lungs are strong competitors for bone-marrow stem cells (main source of muscle and adipose progenitor cells) which is required for cell regeneration of peripheral tissues. Greater than 60% of bone marrow derived stem cells are used by the lungs (Rochefort et al., 2005) for regenerating the short-lived airway epithelial cells (Murphy et al., 2008; Rawlins and Hogan, 2008). This suggests a much higher demand of the lungs for carbon and nitrogen against other tissues. Acute airway epithelium damage induced by acute ventilations during aerobic exercise significantly increases phagocyte infiltration to the lungs (Adams et al., 2011; Leibacher and Henschler, 2016; Combes et al., 2019). This also induces cell regeneration following phagocytic clearance of unhealthy cells in airway epithelial lining in a way similar to muscle inflammation (Su et al., 2005). Massive consumption of bone marrow immune cells and stem cells by the lungs may explain why high-intensity aerobic exercise has greater magnitude of fat loss effect compared with resistance exercise (Willis et al., 2012).

## Alternative Theory

During unfed conditions, visceral adipose tissues continuously releases fatty acids into circulation (Coppack et al., 1990), together with normal turnover of adipocytes and endothelial cells in adipose tissues (Spalding et al., 2008). Therefore, post-meal carbon and nitrogen returning to fat cells determines the abdominal fat mass (Coppack et al., 1990; Chen et al., 2006). Skeletal muscle is a competitor for the post-meal carbon and nitrogen (Ivy et al., 1988) and therefore decreases post-meal carbon and nitrogen returning to adipose tissue. Increasing muscle demand at the time when post-meal nutrients are supplying into circulation can minimize the substrates returning to adipose tissue. This concept is supported by animal and human studies in which providing meal immediately after resistance training results in greater magnitude of muscle mass gain and fat mass loss compared with the condition of detaching mealtime away from the workout (Suzuki et al., 1999; Cribb and Hayes, 2006).

Studies employing dual energy X-ray absorptiometry have also provided solid support for the carbon and nitrogen redistribution effect of exercise training by the evidence of concurrent increases in lean body mass and decreases in fat mass (Cribb and Hayes, 2006; Abbasi, 2019; Kemmler et al., 2021). This nutrient redistribution effect remains noticeable in sarcopenic elderly aged above 70 years and above (Kemmler et al., 2020) and perimenopausal women (Coll-Risco et al., 2019). It is likely that the decreased abdominal fat accumulation after exercise training is associated with increased muscle regeneration attracting more postprandial carbon and nitrogen to challenged muscle tissue against abdominal adipose tissues (Huang et al., 2017; Tidball, 2017). Interventions that promote muscle growth have been shown to decrease fat mass (Mcpherron and Lee, 2002; Leong et al., 2010). Muscle regeneration during inflammation is known to contribute muscle mass gain (St Pierre and Tidball, 1994). High-intensity exercise causes immune cell infiltration into challenged muscle to eliminate senescent cells (Huang et al., 2017; Yang et al., 2018). The inflammation process includes stem cell homing, proliferation, and differentiation after phagocytosis by infiltrated immune cells in challenged muscle tissues (Tidball, 2017; Wu et al., 2019). The increased reconstruction demand of exercised muscle may partly explain the disparity in the development of muscle and adipose tissues after exercise training.

Lipoprotein lipase (LPL) attached on the surface of endothelial cells in capillary lumen determines relative partition of circulating triglycerides to muscle and adipose tissues after meals. The molecular size of triglyceride carried by chylomicron and VLDL is too large to transport across cell membrane of adipocytes from blood unless it is locally hydrolyzed by LPL in the adipose and muscle tissues. Relative LPL expression in adipose tissue and muscle tissues thus determines the daily distribution of circulating triglycerides (chylomicron and VLDL) partitioning into adipose tissues and skeletal muscle after meals. This ratio is substantially influenced by exercise training, in which trained women have relatively higher (∼8 times) muscle-to-adipose tissue LPL ratio compared to their untrained state (Simsolo et al., 1993). This suggests that exercise training favors postprandial triglyceride partitioning into skeletal muscle rather than adipose tissue.

## Concluding Remarks and Future Perspective

Fatty acids (from lipolysis) are continuously released from abdominal adipose tissue into the circulation and fat cells are continuously dying in normal human adults. The size of adipose tissue is determined by the magnitude of nutrient competition from muscle and lungs for cell regeneration and energy replenishment after exercise. This is varied by types of exercise (aerobic or resistance exercise). Despite the fact that lower exercise intensity relies more on fatty acid oxidation, high-intensity exercise training (anaerobic in nature) provides a superior abdominal fat loss effect than low- and moderate-intensity exercise training. Given the fact that exercise does not increase 24-h fatty acid oxidation during and after exercise training, the carbon and nitrogen redistribution theory is more suitable to explain the abdominal fat loss outcome of exercise training than fat burning theory. This reasonably explains why low- and moderate-intensity exercise often fail as strategies for fat loss despite the greater percentage of fatty acid oxidation compared with high intensity exercise. Studies on inter-tissue communication during exercise (such as muscle-derived extracellular vesicles) for post-meal carbon and nitrogen redistribution are promising and may provide useful application to normalize body composition and prevent obesity. Furthermore, the role of fatty acids on repairing post-exercise damage deserves further investigation. More data are needed to support the carbon and nitrogen redistribution theory on fat loss effect of exercise.

## Author Contributions

Both authors contributed significantly to this work.

## Conflict of Interest

The authors declare that the research was conducted in the absence of any commercial or financial relationships that could be construed as a potential conflict of interest.
